# COVID-19 in China: A Rapid Review of the Impacts on the Mental Health of Undergraduate Students

**DOI:** 10.3389/fpubh.2022.940285

**Published:** 2022-06-29

**Authors:** Hairong Shi, Haixue Zhu, Yan Ni

**Affiliations:** ^1^School of Biology and Food Engineering, Chuzhou University, Chuzhou, China; ^2^School of Educational Science, Chuzhou University, Chuzhou, China; ^3^School of Marxism, Nanjing Normal University, Nanjing, China

**Keywords:** COVID-19, mental health, China, strategies, undergraduate

## Abstract

Public health crises pose challenges for governments and health systems, and the coronavirus disease 2019 (COVID-19) has presented major challenges to humans worldwide. In the context of COVID-19 in China, we explore the impacts of the pandemic on the mental health of undergraduate students. We examine pandemic prevention and control measures in Chinese universities through a rapid review and use our findings to explain the difficulties that undergraduate students face. Moreover, our analysis examines the impacts on five aspects of mental health: emotional aspects, personality, interpersonal relationships, learning behavior and employment options. Additionally, we provide implications in four areas based on the application of the study: strengthening psychological intervention, promoting government information disclosure, improving family communication and adjusting self-awareness.

## Introduction

The novel coronavirus was first identified in Wuhan, China, in December 2019 and can cause a respiratory infectious disease called coronavirus disease 2019 (COVID-19) (1). Since the beginning of 2020, COVID-19 has spread rapidly around the world. 2022 is the third year of the global spread of the COVID-19 pandemic, and mutant strains have appeared one after another (2, 3). Large-scale vaccination has continued to be promoted, but the pandemic continued to recur, and the number of infected cases and deaths continued to increase (4). To date, data show that the number of confirmed infections in the world has exceeded 500 million, and the death toll has exceeded 6 million. According to data from Johns Hopkins University 14, the country with the most confirmed cases is the United States, with more than 80 million cases, accounting for nearly one-third of the global total. The United States is also the country with the most deaths, with 1 million deaths thus far. Countries with a relatively high number of confirmed infections also include India, Brazil and France, with 43 million confirmed cases in India, 30 million in Brazil and 28 million in France. Today, there are traces of COVID-19 almost everywhere in the world (4).

Globally, some countries (regions) are still in a state of emergency. Strict pandemic prevention measures are being adopted or maintained to intensify the prevention and control of the COVID-19 pandemic. In some countries (regions), the severity of the COVID-19 pandemic has lessened, and some restrictive measures have been gradually relaxed (4–6).

**Figure 1:**
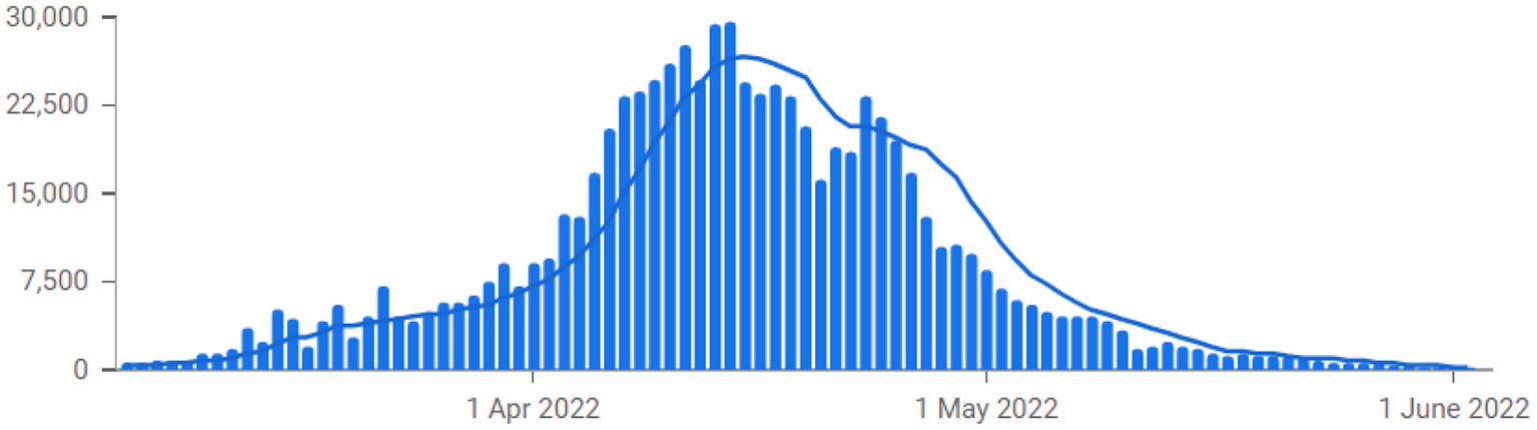
COVID-19 cases from March to June 2022 in China. Data source: our world in data.

After the outbreak of the Wuhan pandemic, China quickly introduced various pandemic prevention policies and measures to bring the pandemic under control. The pandemic situation in China has been under control domestically (7). However, recently, due to the influence of the mutated Omicron virus, the local pandemic in China has rebounded. Local diagnoses continue to occur in Guangdong, Shanghai, Jiangsu, Jilin and other provinces. Since March 2022, the overall number of infections in the country has increased sharply (8, 9). From March 1–24, the cumulative number of local infections reported nationwide exceeded 56,000, affecting 28 provinces (Figure 1). Among these, Jilin Province still has a high level of infections. From March 1–24, a total of more than 29,000 cases of infection were reported. With more than 1,000 new infections per day for several consecutive days, the pandemic situation in Jilin City and Changchun City is continuing to develop (4). The severity of the pandemic in Shanghai, Hebei Province, Fujian Province, and Liaoning Province has grown rapidly in recent days. The risk of community transmission at each of the outbreak sites persists. The pandemic situation in Qingdao, Weihai, and Zibo in Shandong Province and Shenzhen and Dongguan in Guangdong Province was initially controlled (10–12). The pandemic situation in Beijing, Chongqing, Zhejiang Province and other places has stabilized. In terms of vaccinations, China had reported a total of 3 billion doses of the COVID-19 vaccine by end of May 2022. The total number of people vaccinated reached 1.2 million, and 90% of the country's total population has been fully vaccinated.

In this research, we will explore the impacts of COVID-19 control strategies on the mental health of undergraduate students in China. Four sections follow this introduction. Section pandemic prevention and control strategies in Chinese universities and the difficulties faced by undergraduate students introduces China's COVID-19 control strategies in universities and the difficulties that undergraduate students face. Section the impacts on the mental health of undergraduate students caused by the pandemic states the impacts of the pandemic on the mental health of undergraduate students. Section implications provides implications for different parties to improve the mental health of undergraduate students. Section conclusion concludes the paper.

## Pandemic Prevention and Control Strategies in Chinese Universities and the Difficulties Faced by Undergraduate Students

### Pandemic Prevention and Control Measures in Chinese Universities

Recently, many places in China have taken a series of measures to curb the spread of the pandemic, such as allowing work from home, prohibiting the cross-regional movement of people, and the closed management of undergraduates and universities (1). With the development of the internet, work tasks for an increasing number of industries can be performed online (2). Therefore, most companies in closed areas adopt the method of letting employees work from home to maintain the normal operation of the company. To reduce the spillover and spread of the pandemic caused by interprovincial and interregional movement, Jilin Province banned the interprovincial and interregional movement of its own personnel on March 14, 2022. If people have special circumstances that require them to leave the province or region, they need to register at the local police station. After returning, they are quarantined and controlled according to relevant regulations on pandemic prevention and control. If there are any violation of the above provisions, the relevant departments are be held accountable according to law and discipline. Dongguan Pandemic Prevention and Control Headquarters of Guangdong Province issued a notice on March 14, 2022 that Dongguan city would suspend all non-essential movements. In addition to ensuring urban operation and the transportation of goods and goods supplied to Hong Kong, public transportation and subway service in Dongguan have been temporarily suspended. The communities and villages throughout the city are required to implement enclosure management, retain necessary entrances and exits, and set up inspection points. Checkpoints are open 24 h a day, and site codes are required for entry. The city's residents are not allowed to leave Dongguan unless it is necessary (6). Anyone with special needs will leave Dongguan with a 24-h nucleic acid negative certificate. In addition to ordinary high school boarding students who are in their senior year, offline teaching has been paused for students at all levels and types of schools in the city (including children in kindergartens). All kinds of training institutions and custodial childcare institutions have suspended offline training and custodial childcare services 22. Shanghai Fudan University announced that campus closure management will begin at 20:00 on March 13, 2022, and teachers and students will not leave the school. Teachers and students in the school will carry out nucleic acid testing as needed. The campus is located in a separate area behind the school's gate and implements relatively closed management. Teachers and students do not move across.

**Figure 2:**
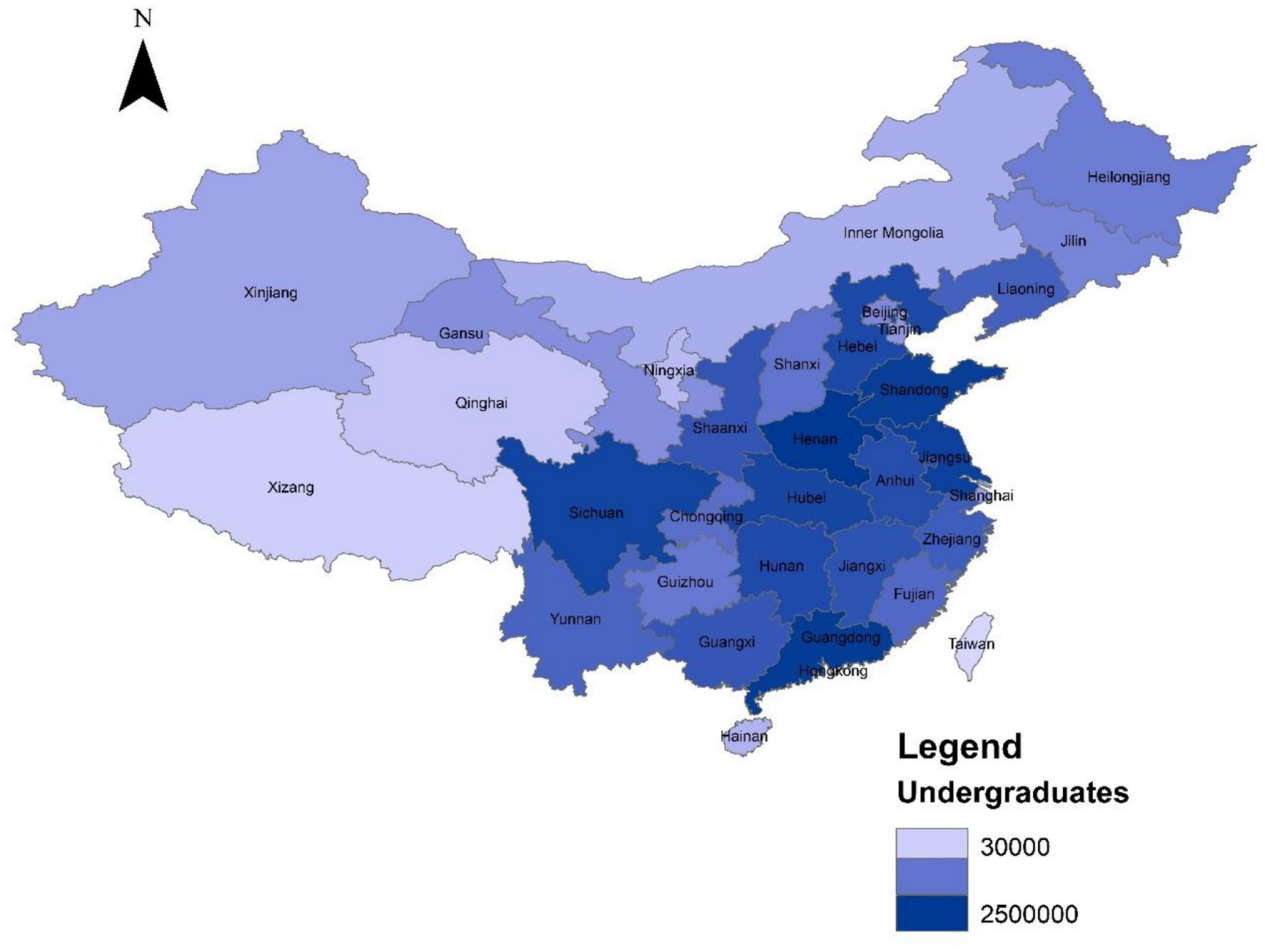
Distribution of undergraduates in China. Source: collected from Ministry of Education of the People's Republic of China and designed by the authors. The darker the color, the greater the number of undergraduates, and vice versa.

China has more than 30 million undergraduate students (Figure 2 and Table 1). In the face of the pandemic, the specific prevention and control measures implemented by Chinese universities mainly include (1) daily temperature monitoring, (2) protective measures for dining in canteens and (3) comprehensive online teaching.

**Table 1:** Number of regular students for normal and short-cycle courses in higher education.

**Province**	**Undergraduates number**	**Provincial population**	**Percentage of provincial population (%)**
Henan	2,492,185	99,365,519	2.51
Guangdong	2,400,227	126,012,510	1.90
Shandong	2,291,483	101,527,453	2.26
Jiangsu	2,014,698	84,748,016	2.38
Sichuan	1,800,903	83,674,866	2.15
Hubei	1,616,873	57,752,557	2.80
Hebei	1,604,798	74,610,235	2.15
Hunan	1,510,332	66,444,864	2.27
Anhui	1,368,465	61,027,171	2.24
Jiangxi	1,241,984	45,188,635	2.75
Shaanxi	1,210,048	39,528,999	3.06
Guangxi	1,184,167	50,126,804	2.36
Zhejiang	1,148,737	64,567,588	1.78
Liaoning	1,140,799	42,591,407	2.68
Yunnan	964,205	47,209,277	2.04
Fujian	947,187	41,540,086	2.28
Chongqing	915,556	32,054,159	2.86
Shanxi	841,986	34,915,616	2.41
Guizhou	840,249	38,562,148	2.18
Heilongjiang	825,601	31,850,088	2.59
Jilin	726,957	24,073,453	3.02
Beijing	608,866	21,893,095	2.78
Gansu	581,062	25,019,831	2.32
Tianjin	572,152	13,866,009	4.13
Shanghai	540,693	24,870,895	2.17
Xinjiang	486,680	25,852,345	1.88
Inner Mongolia	486,647	24,049,155	2.02
Hainan	230,062	10,081,232	2.28
Ningxia	146,679	7,202,654	2.04
Qinghai	74,111	5,923,957	1.25
Tibet	38,556	3,648,100	1.06

*Data source: collected from Ministry of Education of the People's Republic of China. http://www.moe.gov.cn/jyb_sjzl/moe_560/2020/gedi/202108/t20210831_556496.html*.

#### Body Temperature Monitoring

Undergraduates and universities have implemented the system of daily temperature reporting and zero reporting and have set up temperature monitoring points at the school entrance (8). All students must have their temperature taken and submit a health code. Only those with normal body temperatures can enter the campus. Students with body temperatures of 37.3°C and above are not allowed to enter the campus (1). Arrangements will be made to isolate students at a temporary isolation point outside the school. The counselors or class cadres in the school conduct temperature checks on the students every morning, midday, and evening. Temperature monitoring posts are also set up in crowded places, such as classroom buildings, libraries, canteens, and dormitories, to monitor the temperature of those entering (3). During the testing process, if a student's body temperature reaches 37.3°C (inclusive) or above or has symptoms such as dry cough, shortness of breath, muscle aches and weakness, the student counselor will immediately report to the school's leading group for pandemic prevention and control. The emergency plan will be activated, and the students will be quickly sent to the temporary observation places in each building by special personnel wearing masks and then sent to the fever clinic of the designated hospital for further examination (10).

#### Pandemic Prevention for Dining in Canteens

During the pandemic, most undergraduates and universities have required students to wear masks when entering the canteens. In addition, the staff must show the health code and itinerary code. In the temperature measurement area at the entry, the body temperature will be detected by an automatic infrared thermal imaging thermometer (5–7). Those with abnormal body temperatures will be sent to the outdoor observation and remeasurement area for remeasurement or treatment. Hand sanitizer will be used to disinfect after the temperature measurement. The canteen implements single-person, single-seating, with students sitting in the same direction (8). The school encourages students and teachers to eat meals at different times and to eat outside the canteen. If the number of people indoors reaches capacity, the canteen will take temporary measures to restrict the flow. When queuing to pick up meals, people will need to line up along a yellow line on the ground and pick up meals in an orderly manner at the window (13).

#### Online Teaching Mode

The COVID-19 pandemic continues unabated. To actively respond to the impact of the COVID-19 pandemic on classroom teaching, the Ministry of Education put forward the overall plan for deployment and set requirements to allow students to continue teaching and learning while suspending in-person school (14, 15). Due to the impact of the COVID-19 pandemic since the beginning of 2020, schools nationwide have postponed the school enrolment date. Undergraduates and universities across the country have begun online teaching on a large scale. A total of 265 million current students have largely switched to online courses (16–18). Data show that during the pandemic, the number of daily active users of multiple online education applications reached more than 10 million. Among them were 22 online course platforms that were launched by the Ministry of Education, offering 24,000 online courses (19). The online course offerings have provided a guarantee for ordinary undergraduates and universities to be able to suspend in-person classes but continue teaching during the pandemic (20). A number of office applications have been used for cross-border online education. Office applications such as DingTalk and Tencent Conference have become online education platforms and are widely used by teachers and students across the country. At the same time, Huawei has also joined the online education industry and has launched online education classrooms or teaching systems. At present, the online teaching environment includes the following three types: First, online teaching resources and platforms, such as Ai Course (Chinese University MOOC). The second is self-service live broadcast platforms that are accessed from home, such as Zoom, Tencent Conference, DingTalk, etc. This kind of platform is simple and easy to operate, and students and teachers can also communicate and provide feedback on their learning progress in real time. This model is very close to the environment of offline classrooms. The third is an online teaching management exchange platform at home. WeChat and QQ can be used for online class management and communication and arrange, supervise and evaluate the self-learning situation of class students. According to relevant surveys (21, 22), most of the undergraduate students expressed their approval for the online teaching carried out by undergraduates and universities. This shows that online learning works well. A total of 21.9% of undergraduate students strongly agreed with the current online teaching method used by teachers and believed that the learning effect was good and that the results were great. A total of 32.99% of students agreed with online teaching and thought that online learning was more effective. A total of 25.33% of students believed that methods used for online teaching were conventional, and the learning effect was similar to offline teaching. However, 18.48% of the students did not agree with the online teaching method currently used by teachers and believed that the learning effect was not as good that provided by offline learning (21–23).

### Difficulties That Undergraduate Students Face

Undergraduate students have many different characteristics from other groups that are affected by the pandemic. First, undergraduate students have just entered the stage of early adulthood. Compared with other younger students, undergraduate students already have certain knowledge and abilities (10). They have already reached a certain level in terms of information acquisition and comprehension and the channels they use for communication. Relative to other age groups, social network media is used the most widely by undergraduate students (24). During the pandemic, undergraduate students have been studying at home, and they spend more time online than usual. In addition, various news items about the pandemic on the internet can be overwhelming because they occupy so much of various social platforms, and it is almost impossible for people to ignore this information. Receiving relevant information from various media reports every day has also led to a great increase in the fear and anxiety of undergraduate students (23).

Second, according to relevant research, at the physiological level, the development of the physiological functions of undergraduate students is basically mature. However, the frontal lobe, the area of the brain responsible for emotion and control, is not yet fully developed (25). Therefore, undergraduate students still have a relatively weak psychological capacity and ability for the resolution of emotions (26). They are emotionally sensitive and unstable. This causes undergraduate students to suffer from greater emotional fluctuations when facing emergencies such as major pandemics, and therefore, they are more likely to cause damage to their social functions (23, 24).

In addition, undergraduate students have considerable abstract thinking ability and have begun to rationally judge and understand things. However, their way of thinking is more subjective and one-sided (27). This conflict in thinking ability can easily lead to extreme thoughts in the face of major pandemics and even to adverse consequences as a result. Therefore, due to confrontation with the novel situation of pandemic prevention and control, the mental health of undergraduate students still faces huge impacts and challenges (28).

## The Impacts on the Mental Health of Undergraduate Students Caused by the Pandemic

The impacts on the mental health of undergraduate students mainly include emotional aspects, personality, interpersonal relationships, learning behaviors and employment options. Below is the detailed review of those five aspects. It's worth to note that all the numbers we use are reported from Chinese previous studies.

### Emotional Aspects

Anxiety is an emotional reflection of a person's serious deterioration in the value characteristics of real or future things. This includes anxiety, tension, fear and other elements. It is associated with critical situations and unpredictable and unmanageable events, and anxiety may be relieved after the situation changes. According to a survey during COVID-19 (29), the median scores of undergraduate students' anxiety and depression were 2.00 (1.00, 5.00) and 1.00 (0.00, 4.00), respectively. According to the scoring standard, 2,849 people (73.41%) had no anxiety. The numbers of undergraduate students with mild, moderate and severe anxiety were 900 (23.19%), 105 (2.71%) and 27 (0.70%), respectively (28–30). A total of 3,060 people (78.85%) had no depression, and the number of undergraduate students with mild, moderate and severe depression was 659 (16.98%), 123 (3.17%), and 39 (1.01%), respectively. Spearman rank correlation analysis showed that the correlation coefficient between GAD-7 anxiety scores and PHQ-9 depression scores was 0.56 (*P* < 0.00). This shows that the depression and anxiety of undergraduate students are highly positively correlated under the stress of the COVID-19 pandemic. In order to prevent the escalation of the pandemic, universities have postponed the start of school. Undergraduate students have had to reduce their frequency of going out, which has prevented them from attending school and participating in social activities normally (30). This may affect their learning progress and exacerbate their anxiety and depression. Therefore, the mental health problems of undergraduate students cannot be ignored. Among the 3,881 undergraduate students surveyed, the incidences of anxiety and depression were 26.60 and 21.16%, respectively. It is higher than the survey for undergraduate students under normal circumstances (25, 26). It is evident that under the stress of the COVID-19 pandemic, the incidence of anxiety and depression in undergraduate students has increased significantly. It is urgent to take measures of targeted psychological intervention and provide health education.

### Personality

Personality is unique to an individual and different from others (14). Its formation is linked to the external environment and congenital conditions. As a relatively stable psychological factor, it determines an individual's cognition and behavior. It is an important psychological basis for coping with stressors. Personality characteristics such as suspiciousness, sensitivity and timidity affect an individual's cognitive evaluation of things (16, 17). Personality can create unrealistic psychological conflicts, setbacks and other psychological stimuli, preventing people from choosing effective coping methods to eliminate the psychological stress response (31–33). It is mentioned in the social cognitive theory that mental health problems caused by stress are more likely to occur in people who choose to escape psychologically and regard the stressors as a long-term catastrophic threat. Studies have shown that an optimistic personality is conducive to the maintenance of health, and the disease morbidity and mortality are lower in optimistic individuals than in than those who have a pessimistic personality. It also has a strong psychological function and helps an individual maintain a good psychological state and positive emotions. Under the pandemic, because most undergraduate students' communication with each other has been limited to social tools and massively multiplayer online games mediated by the internet, there has been a lack of sufficient offline communication (31). At the same time, the frequency of communication between classmates who have no close relationship is almost zero. As a result, the exchange of communication between undergraduate students and their classmates and communication with those who are not closely related is very scarce. Most of the undergraduate students have experienced a gradual change in their personality traits to a certain extent because they have been alone for a long time or because they have rarely interacted with the outside world, especially with their peers (32).

### Interpersonal Relationships

The quarantine has resulted in a reduction in students' social interaction and interaction with classmates, friends, and teachers (33). Long periods of interpersonal alienation can trigger loneliness in students, resulting in psychological and behavioral biases. An unwillingness to express oneself may be a sign of serious psychological problems such as disharmony with others, inattention to study, addiction to the internet, and even lead to a sense of emptiness (34, 35). According to a relevant questionnaire, 27.21%. of undergraduate students experienced problems getting along with classmates and friends under the pressure of the pandemic. During the pandemic, undergraduate students could not get along with their classmates and friends as they would normally, and the scope of communication was limited (36). They had lived and studied at home alone for a long time. Due to a lack of interpersonal relationships, they have experienced loss, depression, suspicion, and even self-isolation and loneliness, resulting in psychological obstacles. Additionally, they have experienced challenges to interpersonal sensitivity. The troubles caused by interpersonal sensitivity to students are mainly reflected in the fact that they have been with their families since the beginning of control measures for the pandemic. They have had less and less communication, and conflicts have arisen because of some trivial matters (37, 38). Current undergraduate students were born in the internet age, and mobile phones are the most common medium that they use to make friends, shop, obtain information and spend time on the internet. Therefore, they tend to frequently use their mobile phones to watch news or chat, leading to a lack of positive communication with parents. Faced with this situation, many parents often resort to scolding (39). Students feel that it is difficult for their parents to communicate with them, and there is a gap between them, which leads students to think that their parents cannot understand them. Interpersonal relationships are an important part of undergraduate students' daily study and life. After a public health emergency, establishing good interpersonal relationships with relatives, teachers, friends, and classmates is beneficial to the timely relief of psychological pressure. It could improve the understanding of others and help undergraduate students obtain more social support, thereby minimizing anxiety and the incidence of mental health problems such as fear (40).

### Learning Behaviors

During the COVID-19 pandemic, most students have been able to make a study plan and execute it successfully (41). A total of 31.39% of the undergraduate students could make a relatively complete study plan by themselves. A total of 61.09% of the students were able to study using a teacher's plan (42). However, 7.52% of the students still had no study plan. Statistics show that there are gender differences in the study plans and autonomous learning abilities of undergraduate students. A total of 35.51% of the boys said they made a relatively complete study plan, while only 27.55% of the girls said they could study independently according to their own study plan. At the same time, 43.27% of the girls said that they basically studied according to the teacher's plan, while only 31.61% of the boys studied according to the teacher's plan (24). However, there is little difference in the proportion of boys and girls who do not have a study plan. There are also students who are not comfortable with this form of online teaching. During the pandemic, the time scheduled for work and rest has become arbitrary. Due to the irregularity of life and the lack of external constraints, it has been easy to become addicted to games and blur the concept of time. If this condition persists for a long time, it can lead to changes in behavior such as procrastination and irritability (28–30). In addition, for some students, the degree of self-consciousness of learning is not enough, and the accuracy and proficiency of knowledge mastery are insufficient. During the pandemic, online learning forms have lacked the direct supervision and guidance of teachers, which has further affected students' knowledge mastery. In particular, certain training courses that require specialized equipment cannot be practically operated in an online class, and lack the targeted guidance of teachers. This will have a major impact on many students. Therefore, after the resumption of school, many students may have a sense of fear about tests and examinations, and they may be worried about a decline in grades and the possibility of failing assessments. This can even produce anxiety and various physical and mental problems (35).

### Employment Options

According to a survey of graduates on job-seeking behavior (36), when asked if they had started job hunting as a graduating class, among the 1,775 graduates who participated in the survey, 737 had already started job hunting, accounting for 41.52% (25–27). The number of people who had not started job hunting was 1,038, accounting for 58.48% of the graduates. In the process of job hunting, the graduates described their moods (based on 737 people who had started job hunting) with keywords such as nervousness, resignation, etc. (28). For the reasons for not starting a job search (based on 1,038 people who had not yet applied for a job), the graduates selected other keywords, describing reasons such as further education, entrepreneurship, joining the army, preparing for competitions, participating in training, etc. As for the reason they did not want to find a job, some people felt that they could not meet the requirements of the ideal work enterprise. Others thought the job search process was too complicated (32). Some people had not determined what kind of work they wanted to do. Some others stated that it was difficult to find a satisfactory job. In addition, other reasons included choosing between further education and employment, choosing to enter graduate school, being unable to settle down to study, the difficulty of choosing employment, and not yet being mentally prepared (11, 12).

After more than 2 years of the pandemic, the economic growth rate has slowed down, both internationally and domestically (14–16). This has largely affected the overall situation of the Chinese economy. The employment situation of graduates is affected by the impact of the pandemic on the economy, and the employment situation of this year's graduates is extremely severe (6, 7). The pressure on graduates has increased sharply, and many students have chosen to continue their studies to relieve the pressure of employment, resulting in a sharp increase in the number of applicants for entrance examinations (8). The number of applicants for postgraduate entrance examinations in 2022 was as high as 4.57 million, an increase of 800,000 compared with 2021. The enrolment number is 1.107 million, which means that 3 million people will be dropped from the list and will continue to face pressure (37). According to the latest data from the Ministry of Education, in 2022, the number of graduates at the undergraduate level is expected to reach 10.76 million, a year-on-year increase of 1.67 million (34). This is the first time that the number of graduates at the undergraduate level has exceeded 10 million, and it is also the largest increase of the most recent years. The scale and increment of graduates both hit a record high (29, 30).

## Implications

Impacts of COVID-19 on mental health of different group have been explored. The elderly generally experienced significantly lower levels of psychological symptoms including depression, anxiety, and perceived stress. Pregnant women, patients with chronic diseases, and patients with pre-existing severe mental disorders showed mixed results according to each mental health outcome (43). Across countries, the mental health of unemployed people and those experiencing financial insecurity was worse than that of the general population (44). 44.3% of parents with children <18 years living at home reported worse mental health as a result of the COVID-19 pandemic compared with 35.6% of respondents without children <18 living at home (45). Study also indicates that COVID-19 has a considerable impact on the psychological wellbeing of front-line hospital staff. Results suggest that nurses may be at higher risk of adverse mental health outcomes during this pandemic, but no studies compare this group with the primary care workforce (46).

Because the COVID-19 pandemic has not yet ended, the government, universities, and families need to pay close attention to the physical and mental health of undergraduate students. Interpersonal issues, as well as mental symptoms, require appropriate interventions, which may minimize the unavoidable psychological impact of the pandemic (31). It is necessary to carry out health education and provide undergraduate students with relevant information on mental health, physical health, academic skills, fitness and other factors that will improve their lives in a timely manner. To alleviate the psychological problems of undergraduate students, it is recommended to start with measures on the following four levels.

### Strengthening Psychological Intervention for Undergraduate Students

Universities should alleviate the psychological problems of undergraduate students through active guidance, psychological intervention and other measures (42). Universities should try to relieve the psychological pressure of undergraduate students, paying special attention to the emergence of vicarious trauma (34). Undergraduate students experience nervousness and anxiety mainly due to their lack of understanding of the pandemic (24, 25). In the face of panic caused by the unknown, universities should take active measures to guide students to cultivate a correct attitude. First, universities should actively guide students to obtain information through correct and official channels (21). Currently, with the rapid development of online self-media, various sources of information are extensive but cannot be guaranteed to be reliable. Universities need to guide students to obtain information through authoritative channels to reduce the negative impact of rumors. Second, online psychological counseling and intervention should be actively carried out (43). In particular, it is necessary to focus on students who are quarantined and students with confirmed cases of the virus around them. It is important to teach them effective ways to relieve tension and anxiety, and regular telephone interviews and online one-on-one consultations can be adopted to reduce students' psychological fear and resistance (34, 35). Third, publicity and education should be strengthened. During special periods, publicity can be carried out through various channels, such as official accounts and online self-media. This is a good way to guide students to learn about the pathogenesis of the virus, its clinical manifestations and preventive measures for the virus, popularize correct professional knowledge, and reduce students' fear of unknown diseases (36).

### Promoting Government Information Disclosure

According to relevant research findings, actively publicizing the country's policies and measures on the COVID-19 pandemic will strengthen undergraduate students' confidence in the country's ability to fight COVID-19 (1, 2). The data show that the stronger the national prevention and control, the more stable the mentality of undergraduate students (44, 45). First, the government should improve its management mechanism for responding to public emergencies. There are always opportunities to improve the government's programs for joint prevention and control, make policy and regulatory information public and improve undergraduate students' cognitive bias toward COVID-19 and reduce the occurrence of excesses (3–5). Second, the government should make full use of various publicity channels to improve the credibility of mainstream media. The relevant government departments can transmit open and neutral information to the outside world through a standardized and orderly communication system, which can effectively alleviate the public's insecurity about crisis events (6). Mainstream media have a considerable degree of authority and credibility, and government departments should use various channels (46). These measures will help maximize the satisfaction of the public's right to know and actively guide the public to treat the COVID-19 pandemic with a calm and rational attitude (47). On the one hand, these measures can combat rumors and prevent the public from panicking (10). On the other hand, they can enhance the credibility of the government and improve the public's confidence in the government's ability to fight the pandemic. In addition, educational departments at all levels should actively respond to relevant policies and issue policy documents to undergraduates and universities in a timely and accurate manner (11).

### Improving Family Communication

In general, negative emotions are very contagious. Parents are in a state of tension and panic every day, and some even view the pandemic with hostility and anger (22–25). These negative energies are more likely to cause anxiety among students. Therefore, first, as mainstays of the whole family, parents should try their best to regulate their emotions and set a positive example to children (48). It is helpful for parents to create a warm and harmonious family atmosphere in order to help their families through this difficult time. If there is a serious psychological disorder in the family, it is necessary to obtain professional psychological support and assistance in time (27). Second, timely expression of difficult emotions, effective relief, and active companionship should be implemented. Due to passive isolation, the radius of the distance we travel outside the house has decreased, which especially causes difficulties for lively and active undergraduate students, who are desperately looking forward to returning to school and resuming interpersonal interactions (11, 12). This indefinite bondage and imprisonment causes undergraduate students to have many negative emotions. If the accumulation of negative emotions is not relieved in time, it will form psychological garbage and cause psychological problems. Parents need to pay attention to the emotional changes of their children at all times and give them appropriate comfort (24). They should also take the initiative to help children reduce stress, such as doing sports together and doing housework to improve their moods and help them maintain positive and optimistic attitudes. In addition, parents should fully understand their children, trust them, and communicate rationally with them. The longer they live together under the same roof, the more conflicts there will be between parents and children (22). Parents should have the courage to face up to their children's personality growth and be willing to accept imperfect children. Parents should also give children some space for independent reflection and respect their choice of views and needs. When encountering a problem, parents should not intensify the conflict, wait for both parties to calm down and then communicate positively (19–21).

### Adjusting Self-Awareness

Students should adapt themselves to the changing environment through intellectual control and strengthening of will (4). First, we should strengthen the ability to judge information. Attention should be given to authoritative information, scientific understanding should be built, and the ability for information discrimination should be improved (15–17). Faced with a large amount of complex information, undergraduate students should not pay too much attention to it in order to avoid psychological overload. They should only pay attention to news information from official and formal channels (20). They should not believe or spread rumors, keep their minds sober, and not lose their abilities to judge due to the influence of the objective environment (21). Second, it is necessary to strengthen personal hygiene protection, improve the awareness and ability of self-protection, actively respond to policies to achieve active isolation, and avoid cross-infection by avoiding crowded areas (25). Students can take measures such as wearing a mask when going out, disinfecting frequently, etc. Students should also remain vigilant. At this stage, the country has achieved a staged victory in the treatment of the pandemic (44). Therefore, while maintaining vigilance, we should also remain calm and trust the medical workers who are struggling on the front line. In addition, students should actively learn about psychological matters, and learn to rationally regulate emotions and correctly understand negative emotions. It is normal for individuals to have some negative emotions after a major event (34). A moderate emotional response is a self-protective measure of human beings. When students experience negative emotions such as fear and tension, it is important for them to take the initiative to communicate with others (23). Although restricting travel has caused great trouble to study and life, it is still necessary to maintain optimism and enhance self-immunity to effectively avoid the spread of viruses (49).

## Conclusion

At present, the global pandemic situation remains severe. The United States, Europe, South America and some countries in South Asia are still seeing a significant increase in new confirmed cases. The death toll is also rising, with anxiety and fear over the outbreak looming over people's minds. Many countries are still unable to escape the suffering caused by the pandemic. The new round of pandemic counterattacks in China is characterized by many points of infection, wide areas and frequent occurrences (25, 49). The pandemic occurred in more than 20 provinces, the number of newly confirmed local cases and asymptomatic infections increased rapidly, and the pandemic has been scattered throughout many places. The scope of the spread has been further expanded. Community transmission in some areas has not been interrupted, spillover cases have been reported, and the pandemic situation is equally severe and complex. The ongoing COVID-19 pandemic has had a huge impact on undergraduate students' study and daily life, disrupting the learning process and daily lives of undergraduate students. At this stage, undergraduate students should make changes to actively cooperate with the strict home prevention and control measures taken by their communities and local governments. It is also necessary for them to work hard to adapt to taking courses at home through live broadcasts. They have many psychological problems in emotion, personality, interpersonal communication, learning behaviors and so on. Emotions such as anxiety and depression have frequently appeared, and there are situations of high anxiety and tension with regards to seeking employment. Faced with this situation, universities need to play an active role to actively guide and help students. Second, the government can play its important role to disseminate relevant information in a timely manner and from a place of scientific authority, which can alleviate psychological problems to a certain extent. In addition, individual students need to enhance their own mental health knowledge. Having good mental health knowledge will enhance the individual's ability to deal with crisis events. Only with the concerted efforts of all parties and the support within our capabilities will undergraduate students successfully persist during them pandemic period and develop a high level of psychological health.

## Author Contributions

HS, HZ, and YN: conceptualization, writing—original draft, and writing—review and editing. HS: funding acquisition and resources. HZ and YN: project administration and supervision. All authors have read and agreed to the published version of the manuscript.

## Conflict of Interest

The authors declare that the research was conducted in the absence of any commercial or financial relationships that could be construed as a potential conflict of interest.

## Publisher's Note

All claims expressed in this article are solely those of the authors and do not necessarily represent those of their affiliated organizations, or those of the publisher, the editors and the reviewers. Any product that may be evaluated in this article, or claim that may be made by its manufacturer, is not guaranteed or endorsed by the publisher.
